# Therapeutic efficacy and patient compliance of levothyroxine liquid and softgel formulations taken with meals: a systematic review

**DOI:** 10.1007/s12020-024-04016-7

**Published:** 2024-08-31

**Authors:** Vittorio Oteri, Salvatore Volpe, Mariarita Lopes, Giulia Sceusa, Andrea Tumminia, Antonino Belfiore, Francesco Frasca, Damiano Gullo

**Affiliations:** Endocrine Unit, Department of Clinical and Experimental Medicine, University of Catania, Garibaldi-Nesima Hospital, Catania, Italy

**Keywords:** Levothyroxine, Hypothyroidism, Liquid, Softgel, Meal, Breakfast

## Abstract

**Purpose:**

Levothyroxine (L-T4) is the drug of choice for treating primary hypothyroidism. L-T4 tablets should be taken at least 30 min before breakfast. Several studies have suggested that serum thyroid profile is not affected by concomitant intake of liquid/softgel L-T4 with meals. Our aim is to review the evidence on therapeutic efficacy and patient compliance with the liquid and softgel formulation of L-T4 taken with meals, also compared with the standard tablet therapy regimen, in hypothyroid patients.

**Methods:**

We performed a systematic review of literature by searching PubMed, Embase, and Cochrane Library databases. PRISMA guidelines were applied, and the risk of bias of the included studies was assessed using the RoB 2 and ROBINS tools. The methodological quality was assessed following the GRADE criteria.

**Results:**

We included 13 studies, accounting for a total of 1697 patients. The timing of liquid L-T4 intake from breakfast did not affect the therapeutic efficacy of the treatment. No significant differences in the absorption of liquid L-T4 were found when administered together with different foods, beverages, drugs, or other supplements. TSH levels are not influenced by taking softgel L-T4 at breakfast; the efficacy of softgel and liquid formulation is similar when they are taken with a meal, but superior to that of tablet formulation. Shifting from L-T4 tablets taken 30 min before breakfast to liquid/softgel formulation taken with the meal improved medication adherence and perceived quality of life of patients.

**Conclusion:**

Liquid and softgel formulation of L-T4 can be taken at breakfast or close to meals, without losing therapeutic efficacy. These formulations could also improve patient compliance and quality of life compared to L-T4 tablet therapy taken 30 min before breakfast.

## Introduction

Hypothyroidism is a common endocrine disorder caused by a lack of thyroid hormone that requires lifelong replacement therapy for the majority of patients. Studies in Northern Europe, Japan and the USA have found its prevalence to range between 0.6 and 12 per 1000 women and between 1.3 and 4.0 per 1000 men [1–4]. European guidelines recommend treating patients <65 years with symptoms suggestive of hypothyroidism and serum TSH < 10 mU/L, as well as those with overt hypothyroidism and advanced subclinical hypothyroidism (TSH ≥ 10 mU/L) [5].

Levothyroxine (L-T4), sometimes in combination with liothyronine (L-T3), is the drug of choice for treating the symptoms and signs of hypothyroidism and maintaining serum thyrotropin (TSH) concentrations within a specified reference range.

Nonetheless, several cross-sectional surveys involving patients undergoing L-T4 have revealed that a significant portion of them is either over- or undertreated [1, 6, 7]. For example, in athyreotic patients L-T4 monotherapy may not provide euthyroidism due to variability in T3 production: indeed, more than 20% of patients in the study by Gullo et al. [8] exhibited abnormal T3:T4 ratios. Many factors can influence the dosage calculation, the absorption, and the effectiveness of L-T4 therapy: physiological, para-physiological, pharmacological, or pathological conditions [9], but also patients related factors such as body weight [10], age [11–13], gender [10, 14], pregnancy [15], compliance [16], etiology of hypothyroidism [17, 18], TSH goal [18], and deiodinase polymorphisms [19, 20].

Furthermore, excipients and variations in L-T4 formulations (tablets, softgel, and liquid solution) might cause variations in absorption rates [21]. After oral administration of the tablet formulation [22–24], 62–82% of the L-T4 dose is absorbed; this absorption occurs within the first three hours of ingestion and is primarily localized in the ileum and jejunum [25]. To avoid alterations in intestinal absorption, L-T4 tablets must be taken on an empty stomach, preferably 60 min before breakfast [26]. However, the drug is typically given 30 min before breakfast to increase patient compliance. Despite this, a large percentage of patients have poor adherence to L-T4 therapy, mostly because the indication of delaying breakfast by at least 30 min is inconvenient and interferes with their lifestyle [27].

It has been stated that the liquid preparation of L-T4, due to its different pharmacokinetics, is able to solve this problem as once the dissolution phase has been overcome, liquid L-T4 is absorbed more quickly by the mucosa of the small intestine [28]. Similarly, a new formulation in pearl-shaped capsules containing L-T4 dissolved in glycerin with soft gelatin as a protective shell has been commercialized with the aim to allow L-T4 to be taken with breakfast [29], without significant loss of adsorption, to increase patient adherence to therapy, and improve quality of life. Reducing or eliminating the amount of time between L-T4 intake and breakfast may increase patient compliance, and, as a result, improve treatment efficacy and reported quality of life.

During the previous decade, several studies have shown that taking liquid or softgel L-T4 with breakfast or beverages does not influence the serum thyroid hormone profile. Several randomized trials have shown that administering the same dose of oral liquid L-T4 at breakfast or while fasting—a circumstance that may increase treatment compliance—has similar effects on the thyroid hormone profile [30, 31]. However, there is no record of a systematic review that summarizes all this information. The purpose of this systematic review is to report, organize, and critically evaluate the most recent information on the therapeutic efficacy and patient compliance of L-T4 liquid and softgel formulations taken with meals in hypothyroid patients.

## Materials and methods

### Literature search strategy

We conducted this review following the Preferred Reporting Items for Systematic Reviews and Meta-Analyses (PRISMA) guidelines [32] and methodological advice from the Cochrane Handbook for Systematic Reviews of Interventions [33]. Our main primary endpoint was to evaluate therapeutic efficacy of L-T4 in liquid or softgel formulations taken with meals, assessed by blood levels of TSH, FT4 and FT3 and pharmacokinetics of L-T4. Our secondary endpoint was to evaluate the compliance and quality of life in these patients, assessed by qualitative and quantitative data collected from patients via interviews or questionnaires.

A comprehensive search was performed on three medical electronic databases (PubMed, Embase and Cochrane Library) up to the 13th of March 2024. To achieve the maximum sensitivity of the search strategy, we combined the terms referring to the following topics: L-T4 liquid or softgel formulations, meals food and beverages, timing, adherence, and quality of life. We tailored searches to individual databases, using appropriate controlled vocabulary indexing and natural language search terms; the full search strategy is available in Appendix 1 of Online Resource. The reference lists of all included articles, previous literature reviews on the topic, and top hits from Google Scholar were reviewed for further identification of potentially relevant studies, to ensure completeness of this review. To avoid overlapping with other ongoing reviews, we first searched on PROSPERO website for any similar review and then registered our review protocol (ID CRD42023387983).

### Selection criteria

Eligible studies for our systematic review included those investigating the therapeutic efficacy and patient compliance or quality of life of L-T4 liquid and softgel formulations taken with meals in hypothyroid patients and reporting all types of outcomes. Pharmacokinetic studies involving L-T4 liquid and softgel formulations taken with meals were also eligible for inclusion.

Primary screening of the titles and abstracts was performed by adding studies of any level of evidence published in peer-reviewed journals written in English. Duplicates, abstracts, case reports, conference presentations, reviews without original data, editorials, and expert opinions were excluded. We also excluded studies in which data were not accessible, missing, without an available full text, or not well reported. On the remaining papers, full text screening was subsequently carried out.

**Fig. 1:**
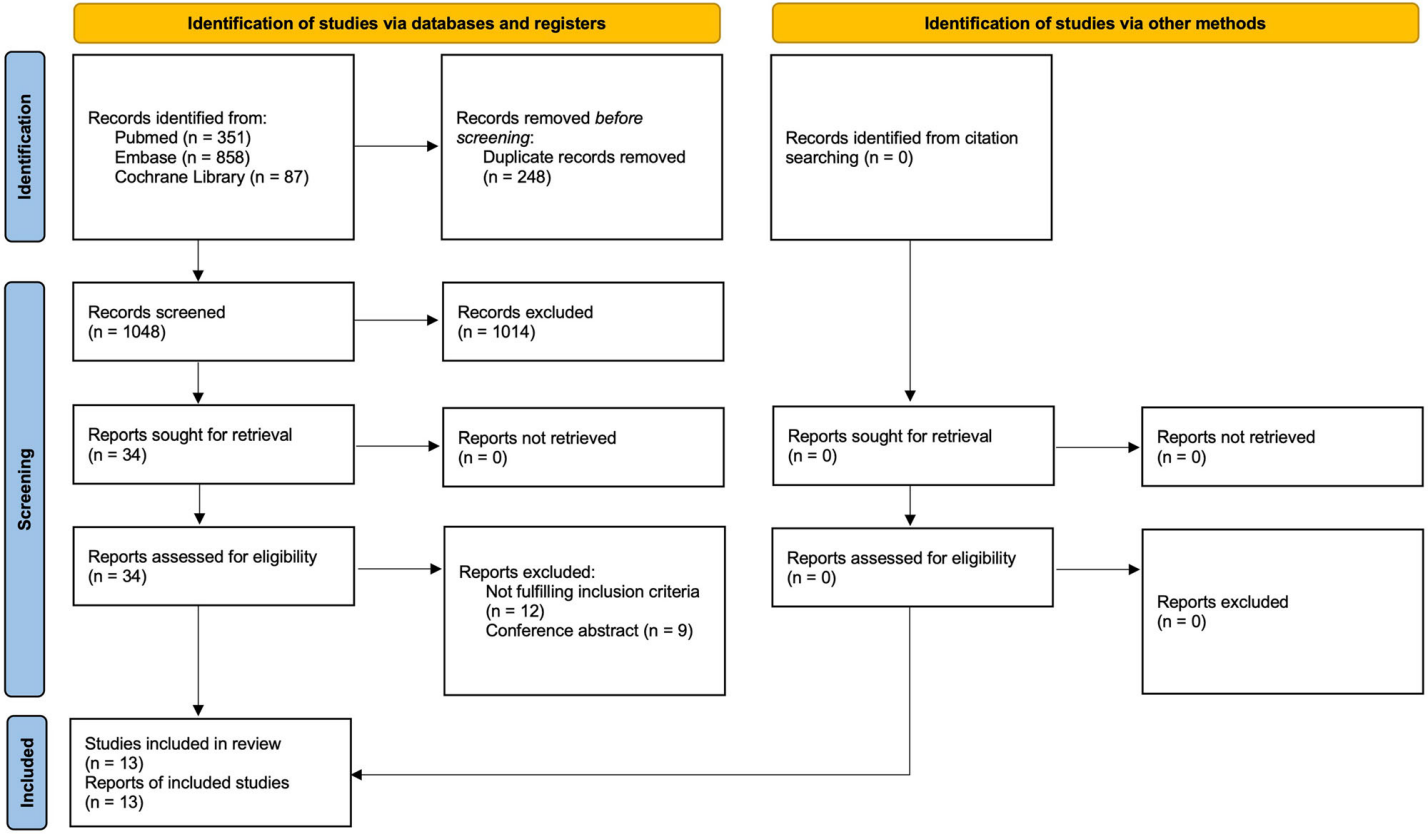
PRISMA flow diagram of study selection

At the beginning of the procedure, each investigator read the abstract or full text of all the articles, selected the relevant ones according to both inclusion and exclusion criteria, and then compared the results with the other investigators, with err on the side of inclusion. After four weeks, the same studies were reread to establish the agreement of the investigators about the articles’ selection. Two authors (V.O. and S.V.) performed the search and evaluated the articles independently. Cases of doubt were solved by consensus with a senior author (D.G.). Figure 1 depicts the PRISMA flow diagram of study selection.

### Data extraction and criteria appraisal

All data were extracted from articles’ text, tables, and figures using the Population, Intervention, Comparison, Outcome (PICO) framework [34] and included at least the following: title, authors, year of publication, country, study design, study population, L-T4 formulation used, associated meal and timing between meal and L-T4 administration, comparator (where applicable), duration of follow-up (where applicable), outcomes, time of outcome assessment, and conclusions. Three investigators (S.V., G.S., M.L.) independently reviewed each article and extracted the data from the full-text articles to Excel spreadsheet organized tables to analyze each study in a descriptive analysis. A second researcher (V.O.) independently double-checked the primary data extraction from each article. The reviewers’ disagreements were settled by debate and consensus. Senior investigator D.G. examined the final results.

### Risk of bias assessment

Risk of bias (RoB) assessment of the full-text of all the studies selected was performed according to the ROBINS-I tool [35] for non-randomized trials, which evaluates seven domains of bias to reach for an overall RoB judgment (Low, Moderate, Serious, Critical), and the RoB 2 tool [36] for randomized trials, which evaluates five (six for crossover trials) domains of bias to reach an overall RoB judgment (Low, Some concerns, High).

Two authors performed the assessment (G.S. and M.L.) independently. Any discrepancy was discussed and solved with an experienced investigator (V.O.). The risk of bias assessment informed the GRADE assessment and complemented the results section. Table 1aS and 1bS in the Online Resource outline the RoB assessment.

### Study quality assessment

We used the GRADE system to assess the study methodology’s quality at outcome level [37]. The design of a study is a significant factor in determining its starting grade; randomized trials produce high-quality evidence. Given the use of the ROBINS-I tool to assess RoB, the starting grade of observational studies was regarded as high-quality evidence, as described by Schünemann et al. [38]. The examination proceeded with a review of five factors that can reduce the quality of the evidence and three elements that can increase it. This resulted in an overall evaluation of a body of data into one of the four grades: High, we are confident that the genuine effect is close to the estimated effect; Moderate, we are moderately confident in the impact estimate, the genuine effect is likely to be close to the estimated effect, but there is a possibility that it is significantly different; Low, our confidence in the effect estimate is restricted, and the genuine effect may be much different from the effect estimate; Very low, we have very limited confidence in the effect estimate, and the genuine effect is likely to differ significantly from the estimate.

Two authors (G.S. and M.L.) did the assessment independently. Any discrepancies were reviewed and resolved with an experienced investigator (V.O.). The GRADE assessment influenced the Summary of Findings table. Table 2S in the Online Resource summarizes the quality assessment.

## Results

### Study characteristics

We included 13 studies which evaluated therapeutic efficacy and patient compliance in patients treated with L-T4 liquid or softgel formulation taken with meals. They account for a total of 1697 participants (73% F), with a mean age of 49 years. Eleven studies were conducted in Italy (85%) [6, 28–31, 39–44], one was conducted in Italy and Switzerland [45], and one in Canada [46]. Five studies were randomized trials [28, 30, 40, 41, 46], six were non-randomized prospective trials [29, 31, 39, 42–44], one was a real-life prospective study [45], and one was a cross-sectional study [6].

Ten studies analyzed the liquid L-T4 formulation [6, 28, 30, 31, 39–43, 46], one study considered both liquid and softgel formulations [45], and two studies analyzed only the softgel formulation [29, 44]. Among the included studies, seven also made a comparison with tablet L-T4 formulation [6, 29, 40–43, 45]. Breakfast was the most common meal associated with L-T4 intake [6, 28, 30, 31, 39, 40, 42–45].

A comprehensive and concise view of the characteristics of the included studies is provided in Table 3S in the Online resource. Table 1 summarizes the main results of this review.

**Table 1:** Summary of results

Outcome	Main results	GRADE quality of evidence
**Therapeutic efficacy of liquid L-T4 taken at breakfast**	The timing of liquid L-T4 intake from breakfast did not affect the therapeutic efficacy of the treatment [28, 30, 31, 39, 41, 42, 45]. No significant differences were found in the absorption of liquid L-T4 when administered together with different foods, beverages, drugs, or other supplements [30, 31, 39, 42]	**Moderate**
**Therapeutic efficacy of softgel L-T4 taken at breakfast**	TSH levels were not influenced by taking softgel L-T4 at breakfast [29, 44, 45]; the efficacy of softgel and liquid formulation was similar when taken with a meal [29, 44, 45], but superior to that of tablet formulation [29, 45]	**Moderate**
**Pharmacokinetic of liquid/softgel L-T4 administered at breakfast**	Liquid and softgel L-T4 formulations taken with a meal had pharmacokinetic properties comparable to tablet formulations taken on an empty stomach [29, 40, 46]	**Moderate**
**Compliance and quality of life of patients**	Liquid and softgel L-T4 therapy taken at breakfast could improve patient compliance and quality of life [6, 28, 30, 42, 43]	**Moderate**

### Therapeutic efficacy of liquid L-T4 taken at breakfast

Six studies evaluated the efficacy of L-T4 liquid formulation taken at breakfast, taking into consideration both the time elapsed between L-T4 administration and the meal, and the type of meal [28, 31, 39, 42, 44, 46].

When treatment efficacy was evaluated based on the patients’ serum TSH values, the timing of liquid L-T4 intake did not significantly affect the therapeutic efficacy of the treatment [28, 30, 31, 39, 41, 42, 45]. Another study found no significative differences in the therapeutic efficacy for the administration of liquid L-T4 at breakfast versus 10 min before breakfast; in a post-hoc analysis, comparing the administration of liquid L-T4 at breakfast or 10 min before versus administration 30 min before breakfast resulted in minimum differences in TSH levels (TSH ratio = 1.23, with definition of equivalence set at 0.8–1.25) that suggested a clinically equivalence between the two treatments [28]. Moreover, two studies showed no significant differences in serum FT4 and FT3 levels when liquid L-T4 was taken at breakfast or in a fasting state [30, 39].

Four studies analyzed the therapeutic efficacy of liquid L-T4 taken at breakfast in relation to the different types of food and beverages consumed during the meal, finding no significant differences in the absorption of L-T4 when it was administered together with different foods, beverages, drugs, or other supplements [30, 31, 39, 42].

Results from two studies highlighted that no influence was induced on TSH and thyroid hormones levels by a variety of meals such as beverage-only breakfast or with solid foods in addition to beverages. Furthermore, in patients assuming concomitant drug treatment (proton pump inhibitors, calcium, or iron supplements) there were no significant changes in the dose of L-T4 administered or effect on the TSH values. Also, no significant effects produced by dietary fiber and soy milk on TSH levels were noted [30, 31]. Cappelli et al. showed a slight increase in TSH levels in patients who assumed liquid L-T4 with coffee versus patients who assumed the same formulation with water at breakfast followed by coffee intake within few minutes (2.9 ± 0.9 vs. 2.3 ± 1.1 mIU/L; *p* = 0.056) [39].

Giusti et al. demonstrated a significant positive correlation (Sr 0.32, *p* = 0.03) between protein intake and FT3 levels under liquid L-T4 treatment at breakfast, but no correlation was found between calorie intake, normal (<4 g) alimentary fiber intake, or nutrients distribution at breakfast and thyroid hormones and TSH levels; moreover, a significant inverse correlation emerged between TSH and alimentary fiber intake at breakfast (<4 g) in patients treated with liquid L-T4 formulations (Sr −0.36, *p* = 0.01) [42].

Three studies compared the liquid and tablet L-T4 formulations showing no significant differences in terms of therapeutic efficacy at different timings of administration [41–43].

Giusti et al. reported no statistically significant differences in TSH and thyroid hormones when patients switched from the treatment with L-T4 tablets to that with liquid L-T4, maintaining the same eating habits at breakfast; of the patients recruited, 68% took liquid L-T4 more than 30 min before breakfast, while 19% took it between 15 and 30 min before breakfast and 13% less than 15 min before breakfast [42]. Guglielmi et al. demonstrated that switching from tablet L-T4 assumed 30 min before breakfast to liquid L-T4 at breakfast did not lead to significant changes in the values of TSH and thyroid hormones [43].

Another study analyzed the differences between tablet and liquid L-T4 formulations in patients with necessity of enteral feeding tube. Liquid L-T4 was administered directly in the nasoenteric tube without the need for an empty stomach, whereas tablets were crushed before administration and enteral feeding was stopped for 30 min before and after L-T4 treatment. No significant differences in TSH, FT3 and FT4 levels before and after L-T4 treatment were observed in the two groups of patients [41].

### Therapeutic efficacy of softgel L-T4 taken at breakfast

Three studies evaluated the efficacy of L-T4 softgel formulation taken at breakfast, compared to both tablet and liquid formulation, and all demonstrated that TSH levels are not influenced by taking L-T4 softgel at breakfast [29, 44, 45].

One study compared L-T4 tablets and softgel treatment in patients thyroidectomized for benign goiter or with Hashimoto thyroiditis; the two formulations were considered taken correctly if a time interval of at least 30 min before breakfast was respected, or not correctly if this interval was not respected. Among patients using L-T4 incorrectly, mean TSH levels were significantly increased in those on tablet therapy [2.30 (0.75–3.78) vs. 1.65 (0.86–2.70) *p* = 0.0029], whereas no difference in TSH levels was observed in those on softgel or liquid formulation therapy [1.24 (0.35–1.95) vs. 1.70 (1.10–2.17) *p* = 0.36]. With proper intake, TSH values were stable in the tablet L-T4 group [1.65 (0.86–2.70) vs. 1.67 (0.51–3.07) *p* = 0.22] but significantly reduced in the softgel or liquid L-T4 group [0.80 (0.22–1.84) vs. 1.70 (1.10–2.17) *p* = 0.004]. Furthermore, patients receiving L-T4 softgel showed significantly lower serum TSH levels after 6 months compared to patients receiving tablet formulation (1.15 (0.30–1.84) mIU/L vs. 1.97 (0.70–3.45) mIU/L, *p* = 0.0033) [45].

A similar study analyzed the TSH serum values profile in patients taking tablet and liquid/softgel L-T4 formulations in relation to coffee consumption, considering the intake at least 60 min before coffee as “proper” and the intake with coffee or less than 5 min from coffee as “improper”. A significant difference in serum TSH levels was shown between proper and improper intake in patients treated with L-T4 tablets (respectively 0.28 ± 0.20 mU/L vs. 1.23 ± 1.47 mU/L, *p* = 0.00028); after switching to softgel capsules, no difference in TSH levels was found between improper and proper intake (0.34 ± 0.30 mU/L vs. 0.41 ± 0.46 mU/L, *p* = 0.90) [29].

Another paper showed no significant differences in TSH levels after switching L-T4 therapy from liquid to softgel (1.9 (0.5–4.0) mIU/L vs. 2.2 (0.5–4.5) mIU/L, *p* > 0.05); however, a slight reduction was reported in FT3 levels (2.7 (2.4–3.3) pg/mL vs. 2.5 (2.4–3.1) pg/mL, *p* < 0.05) and FT4 levels (10.6 (8.6–13.8) pg/mL vs. 9.9 (8.0–13) pg/mL, *p* < 0.001) [44].

### Pharmacokinetics of liquid/softgel L-T4 administered at breakfast

Three studies have shown that liquid or softgel formulations of L-T4 taken with a meal have pharmacokinetic properties comparable to tablet formulations taken on an empty stomach [29, 40, 46].

Marina et al. studied the increase of serum FT4 after a load of 200 mcg of L-T4, in a group of patients thyroidectomized for thyroid cancer. There were no significant differences in the percentage of FT4 increase between the 3rd and 4th hour after L-T4 ingestion in three considered groups: one who assumed L-T4 tablets while fasting, one who assumed liquid L-T4 while fasting and one who assumed liquid L-T4 at breakfast. Also, there were no significant differences in the three groups comparing the maximum percentage of FT4 increase [40]. Another study, which enrolled 33 healthy volunteers aged 18 to 50 years, analyzed the absorption test of a 600 μg load of liquid L-T4 administered 15 or 30 min before a high-fat high caloric meal: the mean serum total T4 concentration-time profiles appeared similar, and the areas under the curve (AUC) in the two groups were substantially equivalent [46]. Vita et al. examined the absorption of a load of 600 μg of L-T4 softgel formulation taken by two volunteer patients under TSH-suppressive treatment with water or with coffee, evaluating blood levels of T4 with serial blood draws up to 4 h after administration. The results showed that the effect of coffee on this type of formulation is negligible, particularly on the maximum concentration peak [47].

### Compliance and quality of life of patients

Five studies analyzed therapeutic compliance and quality of life of patients and evaluated their preferences regarding the timing of administration and formulation of L-T4 [6, 28, 30, 42, 43].

Comparing the intake of L-T4 at least 30 min before or during breakfast, all patients unanimously declared that they preferred taking the therapy at breakfast [30]. In another study, adherence to L-T4 therapy (liquid or tablet formulation) among patients was measured with the eight-item Morisky Medication Adherence Scale (MMAS-8) [48]; the MMAS-8 score subdivides medication adherence into low (<6), medium (6 or 7) and high (>8). The population consisted of 1.9% low adherers (1.2% on tablets and 2.5% on liquid), 10.9% medium adherers (10.6 and 11.3%, respectively) and 87.2% high adherers (88.2 and 86.2%, respectively). The results also showed that patients on tablet L-T4 forgot to take their medication more frequently than those on liquid L-T4 treatment (64.5% vs. 34.5%, *p* < 0.001), felt more hassled about sticking to their therapy (72% vs. 39.6%, *p* < 0.001) and had more trouble remembering to take all their medications (67% vs. 26.2%, *p* < 0.001); these results were confirmed by a subset analysis matching patients one to one in accordance to their age.

In the study by Cappelli et al. the MMAS-8 was also supplemented with 3 items to evaluate preferences between tablet or liquid formulations: 51.6% of patients preferred tablets for lifetime medication therapy, even though taking tablet 30 min before breakfast was a problem for 71.2% of patients; the 77.8% of patients believed that liquid L-T4 ingested with breakfast was the best choice [6].

In the paper by Giusti et al., adherence to therapy was evaluated after shifting from tablets to liquid formulation in patients operated for differentiated thyroid cancer (DTC). It was shown that only 8% of patients discontinued liquid L-T4 because of gastric pain (*n* = 4), and palpitations (*n* = 1). A visual analogical scale (VAS), regarding the general acceptability of L-T4 therapy, ranging from 0 (strong dislike) to 10 (maximum degree of liking) was administered: the tablet formulation proved to be significantly (*p* < 0.001) preferred than the liquid formulation. However, the number of patients reporting subjective complaints was significantly lower on the liquid (28%) than on the tablet (43%) L-T4 formulation (*p* = 0.03). At the final examination, only 27% of patients asked to return to L-T4 tablets formulation because of their palatability (16% of all patients), taste (9%) and subjective side effects (2%); in this regard the percentage of DTC patients who decided to remain on liquid L-T4 was statistically significant (73%, *p* < 0.001) [42]. When QoL was evaluated using a Short-Form 12 questionnaire (SF-12), no significant differences either in the physical or in the mental component score were found in relation to the timing of L-T4 intake (30 min before breakfast, with breakfast or 10 min before breakfast) [28].

Another study that used a dedicated QoL questionnaire (ThyTSQ) [49] highlighted an improvement in the quality of life of most patients with no changes in thyroid function after changing their L-T4 intake from “before breakfast” to “at breakfast”. Of these patients 66.6% reported an improvement in their quality of life, whereas 22.5% of them did not any report substantial change, and only 10.7% complained of a worsening in quality of life. Specifically, quality of life was assessed with the following questions: “How satisfied are you with the current treatment for your underactive thyroid?” (from 0.0% at baseline to 66.6% three months after the shift; *p* < 0.01); “How convenient have you found your treatment to be during the last three months (that is remembering to take the medication)?” (from 38.2% to 93.0%; *p* < 0.01); and “How satisfied would you be to continue with your present treatment and dose?” (from 32.2% to 85.0%; *p* < 0.01) [43].

## Discussion

Our analysis demonstrates that L-T4 treatment with liquid/softgel formulations taken shortly before breakfast or during the meal could be a reasonable treatment strategy in hypothyroid patients; this approach is equally effective as L-T4 therapy in tablets taken 30 min before breakfast, based on serum thyroid hormones levels. L-T4 therapy is typically prescribed before breakfast, which is a vital time of day for many patients; indeed, the time interval traditionally required between L-T4 intake and breakfast has long been a problem in L-T4 replacement therapy, but these new formulations could overcome this limitation. Switching to these other formulations may also be recommended for patients who, despite reaching the predetermined TSH target, have difficulty adhering to the therapeutic regimen.

The advantages of using liquid/softgel formulations over tablets are also evident in terms of the types of foods or other drugs taken at breakfast. In these patients, TSH levels are not affected by the consumption of soy milk, dietary fiber, iron or calcium supplements, or proton pump inhibitors (PPI), making it easier to treat patients who need many concurrent medications. Even multivitamin products, widely used in common clinical practice, can also cause malabsorption, especially those containing ferrous sulfate and calcium carbonate; in this case, it has been demonstrated that the simultaneous intake of these substances does not alter the absorption of liquid and softgel formulations [50].

A study conducted on a specific group of patients fed via nasoenteric tube provided additional evidence of the poor influence of foods on the absorption of liquid L-T4 compared to tablets. The achievement of good thyroid hormonal compensation even in these situations, with L-T4 delivered without interrupting enteral nutrition, supports the conclusion that food does not affect L-T4 liquid formulation absorption [41].

PPI and L-T4 are two common medications that are often used together in the morning. Seng et al., for example, demonstrated how the use of PPIs, which reduce gastric acid secretion, led to lower absorption of L-T4 in the tablet formulation when compared to the softgel formulation, determining that, although the two formulations are “bioequivalent” in physiological conditions, the use of L-T4 softgel is desirable in conditions of reduced gastric secretion [51].

Pharmacokinetics is another important factor to consider when evaluating drug absorption. Loading dosage experiments are used to assess the pharmacokinetic features of different formulations. These tests, while not adhering to traditional protocols use comparable approaches. Although taken with a traditional meal or a high-fat, high-calorie breakfast, liquid L-T4 has pharmacokinetics similar to that of L-T4 tablets taken on an empty stomach [52]. Furthermore, no effect on pharmacokinetics was identified with the concurrent assumption of certain drinks such as coffee as compared to water. A major prerequisite for the proper absorption of L-T4 tablets is the creation of an acidic environment at the gastrointestinal level; coffee, in this regard, could have an impact by decreasing gastric acid secretion. Tests conducted with a high-fat meal reveals that a potential reduction in stomach emptying does not impair the absorption of liquid L-T4 [53]. This finding may be relevant for the use of these formulations in patients with gastroparesis, although more research is needed to confirm this observation.

Finally, we investigated patient adherence to medication and satisfaction with a hypothetical switch from standard tablets to liquid/softgel formulations, a factor that is often overlooked by studies in this field. This is a critical aspect of appropriate therapeutic management. We often emphasize the importance of the doctor-patient therapeutic interaction, but we often neglect the fact that inappropriate drug administration and prescription non-adherence are two of the main reasons of therapeutic failure. It is not uncommon to observe patients who are unable to achieve a satisfactory TSH target despite being prescribed a maximum L-T4 dosage (>2 mcg/kg/day); in this case, one of the possible causes, which is sometimes overlooked, is an incorrect assumption of the therapy or poor patient compliance. Some studies have evaluated patient satisfaction and the underlying possible factors and the reasons for switching treatment [54–56].

Our results clearly show that the timing of L-T4 administration has a significant impact on patients’ therapeutic adherence to treatment. The majority of patients interviewed found it advantageous to be able to take L-T4 during breakfast rather than 30 min before. This finding is unsurprising given the frenetic pace of patients’ social and working lives today, where they frequently complain about forgetting therapy or being unable to wait the traditional 30 min between administration and breakfast. In studies that compare the change from “before breakfast” to “at breakfast” administration, patients’ quality of life improves due to higher satisfaction and a lower likelihood of forgetting the medication. The new formulations, therefore, allow greater flexibility in their use and most likely represent a more comfortable and practical solution for the patient [6].

These findings highlight the need of investigating patients’ therapeutic habits to provide alternative treatments that address their concerns. Interestingly, while the traditional tablet formulation is the treatment of choice for most patients, a greater percentage of patients express complaints about tablet therapy than patients who use liquid/softgel formulations. Patients’ decisions to use tablet therapy are likely motivated more by solid habits than by genuine preferences.

However, we need to analyze why some patients prefer L-T4 tablets over liquid L-T4, with reasons mainly related to taste. This concept suggests that improving palatability of liquid/softgel L-T4 formulations may increase the adherence to these treatments. In fact, studies show that treatment with L-T4 in tablets is still the more common option, despite the numerous problems associated with it.

The higher cost of alternate formulations is another factor that discourages patients from switching treatment, although the new formulations allow greater flexibility and seem to represent a more comfortable and practical solution for the patient. In addition to patient satisfaction with these new formulations, it is useful to evaluate the point of view of specialists who treat their patients with the various L-T4 formulations available on the market. Several surveys were conducted to evaluate the therapy options for hypothyroidism (L-T4 alone, L-T4 plus L-T3, or dehydrated thyroid extract), as well as the type of formulation that may be utilized. These surveys, conducted also in Italy, United Kingdom, and Ireland, revealed that L-T4 alone is the most used therapy for the treatment of hypothyroidism, and tablets are the most common formulation. However, in most circumstances, L-T4 liquid/softgel is the best option for individuals who have difficulty establishing adequate hormonal replacement. The presence of interfering drugs, celiac disease, malabsorption, intolerance to lactose or excipients are among the most common reasons given by survey respondents for selecting these formulations, as is the patient’s inability to take L-T4 on an empty stomach separately from food/drink [57–59].

Given the strength of our findings, it is reasonable for every specialist who treat hypothyroidism to consider L-T4 liquid or softgel formulations also as first-line therapeutic choice, according to patient lifestyle and preference, avoiding reserving the use of these formulations just in case of therapeutic failure with tablets.

Following these considerations, national policymakers may consider providing complete reimbursement of liquid and softgel formulations in addition to tablet L-T4; for example, at the time of writing this manuscript, the cost for L-T4 softgel formulation is still not covered by the Italian National Health Service for people with hypothyroidism.

Correct L-T4 absorption must be one of the main considerations in patients who are unable to establish adequate hormonal compensation. Several studies have shown how medications and concurrent diseases might affect treatment targets. Reduced gastric acidity, for whatever reason, can cause L-T4 malabsorption; this problem can be solved by using innovative alternative formulations, as demonstrated by Santaguida et al. in a comparative study of tablet and softgel in a population of patients with impaired gastric acid secretion [60]. Helicobacter pylori (HP) infection is another common cause of malabsorption in the world population. Several studies have demonstrated that maintaining optimal TSH values in people with this illness require higher doses that if maintained after the infection has been eradicated can result in thyrotoxicosis [61, 62]. Ribichini et al. demonstrated that the liquid formulation, compared to tablets, produced both a greater decrease in TSH values and a greater homogeneity of TSH values in patients with active HP infection; these differences disappeared 9 months after the infection was eradicated [63].

Even lactose intolerance (LI), a very common digestive problem, can cause L-T4 malabsorption and hypothyroidism. Five patients with LI and hypothyroidism treated with a sufficient dose of L-T4 tablets (containing lactose) remained hypothyroid while normalized their TSH levels after switching to a liquid preparation of L-T4 (lactose-free) at the same dosage. TSH levels worsened in three of them after returning to the tablets [64].

Bariatric surgery can also cause malabsorption. Fallahi et al. found that in a population of patients who underwent Roux-en-Y-gastric bypass (RYGB) or biliary-pancreatic diversion (DBP) L-T4 tablet therapy did not achieved euthyroidism, whereas TSH levels normalized after switching to liquid L-T4 therapy [65].

More research is needed to determine the efficacy of liquid/softgel L-T4 treatment when combined with various meals or given at other times of day to give the patient maximum freedom of choice.

The main limitation of this review is the heterogeneity of the included studies: differences in research design, protocols, techniques, assessment time points, and PICOs precluded us from performing a quantitative synthesis through a meta-analysis.

## Conclusion

Our systematic review proves that liquid and softgel formulation of L-T4 can be taken at breakfast or near meals, without losing therapeutic efficacy; this administration schedule could also improve patient compliance and quality of life. The use of L-T4 liquid/softgel formulations has the advantage of providing additional flexibility in situations where L-T4 absorption in tablets may be compromised. Therefore, the new liquid/softgel formulations of L-T4 are particularly useful if patients, for any reason, take L-T4 during a meal, as they guarantee therapeutic efficacy and better patient adherence to therapy.

However, further studies are needed to better investigate the influence of various meals, not only breakfast, on the therapeutic efficacy of liquid and softgel L-T4, or different timing schedules at other times of day to give the patient maximum freedom of choice.

## Supplementary information

Online resource
