# Supplementary material for: Therapeutic efficacy and patient compliance of levothyroxine liquid and softgel formulations taken with meals: a systematic review
Source: Endocrine. 2024 Aug 31;87(1):48–58. doi: 10.1007/s12020-024-04016-7 (PMC11739177; doi:10.1007/s12020-024-04016-7)
Supplement: Supplementary file 1 — Online resource [file 12020_2024_4016_MOESM1_ESM.docx]

**Endocrine**

**Therapeutic efficacy and patient compliance of levothyroxine liquid and softgel formulations taken with meals: a systematic review**

**Vittorio Oteri MD^1*^, Salvatore Volpe MD^1^, Mariarita Lopes MD^1^, Giulia Sceusa MD^1^, Andrea Tumminia MD, PhD^1^, Antonino Belfiore MD, PhD^1^, Francesco Frasca MD, PhD^1^, Damiano Gullo MD^1^**

^1^Endocrine Unit, Department of Clinical and Experimental Medicine, University of Catania, Garibaldi-Nesima Hospital, Catania, Italy

**Correspondence should be addressed:*

Vittorio Oteri, Endocrine Unit, Department of Clinical and Experimental Medicine, University of Catania, Garibaldi-Nesima Hospital, Catania, Italy, email: [research@droteri.it](mailto:research@droteri.it)

**Appendix 1.** Search strategy

PubMed search strategy:

(Levothyroxine OR Thyroxine OR L-T4 OR "Thyroxine"[Mesh]) AND (liquid OR fluid OR gel OR softgel OR solution) AND (breakfast OR "Breakfast"[Mesh] OR Meal OR mealtime OR "Meals"[Mesh] OR dinner OR lunch OR "Food"[Mesh] OR food OR "Beverages"[Mesh] OR Beverage OR timing OR "Treatment Adherence and Compliance"[Mesh] OR adherence OR compliance OR "Quality of Life"[Mesh] OR (Quality of Life) OR qol)

Embase and Cochrane Library search strategy:

(Levothyroxine OR Thyroxine OR L-T4) AND (liquid OR fluid OR gel OR softgel OR solution) AND (breakfast OR Meal OR mealtime OR dinner OR lunch OR food OR Beverage OR timing OR adherence OR compliance OR (Quality of Life) OR qol)

**Table 1aS.** Risk of bias assessment of the included randomized trials following the RoB 2.0 tool

*RoB 2.0 for individually-randomized, parallel-group trials*

| **Author et al. (Year)** | **Randomization process** | **Deviations from intended interventions** | **Missing outcome data** | **Measurement of the outcome** | **Selection of the reported result** | **Overall** |
| --- | --- | --- | --- | --- | --- | --- |
| **Marina et al. (2016)** | Low | Low | Low | Low | Some concerns | **Some concerns** |
| **Pirola et al. (2014)** | Low | Some concerns | Low | Low | Low | **Some concerns** |

*RoB 2.0 for crossover trials*

| **Author et al. (Year)** | **Randomization process** | **Period and carryover effects** | **Deviations from intended interventions** | **Missing outcome data** | **Measurement of the outcome** | **Selection of the reported result** | **Overall** |
| --- | --- | --- | --- | --- | --- | --- | --- |
| **Cappelli et al. (2016)** | Low | Low | Low | Low | Low | Low | **Low** |
| **Ducharme et al. (2022)** | Low | Low | Some concerns | Low | Low | Low | **Some concerns** |
| **Morelli et al. (2015)** | Some concerns | Low | Low | Low | Low | Some concerns | **Some concerns** |

**Table 1bS.** Risk of bias assessment of the included non-randomized studies following the ROBINS-I tool

| **Author et al. (Year)** | **Confounding** | **Selection of participants into the study** | **Classification of interventions** | **Deviations from intended interventions** | **Missing data** | **Measurement of the outcome** | **Selection of the reported result** | **Overall** |
| --- | --- | --- | --- | --- | --- | --- | --- | --- |
| **Cappelli et al. (2014)** | Low | Low | Low | Moderate | Low | Low | Low | **Moderate** |
| **Cappelli et al. (2016)** | Low | Moderate | Low | Low | Low | Low | Low | **Moderate** |
| **Cappelli et al. (2018)** | Low | Low | Low | Low | Low | Low | Low | **Low** |
| **Giusti et al. (2014)** | Low | Low | Moderate | Low | Low | Moderate | Low | **Moderate** |
| **Guglielmi et al.  (2018)** | Low | Low | Moderate | Low | Low | Moderate | Low | **Moderate** |
| **Pirola et al. (2018)** | Low | Low | Low | Low | Low | Low | Low | **Low** |
| **Trimboli et al. (2020)** | Low | Low | Low | Low | Low | Moderate | Low | **Moderate** |
| **Vita et al. (2012)** | Moderate | Moderate | Low | Low | Low | Moderate | Low | **Moderate** |

**Table 2S.** Quality assessment of the included studies following GRADE approach

| **Outcome** | **Risk of Bias** | **Inconsistency of results** | **Indirectness of evidence** | **Imprecision** | **Publication bias** | **Large magnitude of effect** | **Dose-response gradient** | **Plausible confounding** | **Quality** |
| --- | --- | --- | --- | --- | --- | --- | --- | --- | --- |
| **Therapeutic efficacy of liquid L-T4 assumed at breakfast** | Unclear | Undetected | Undetected | Undetected | Undetected | N/A | N/A | No | **Moderate** |
| **Therapeutic efficacy of softgel L-T4 at breakfast** | Unclear | Undetected | Undetected | Undetected | Undetected | N/A | N/A | No | **Moderate** |
| **Pharmacokinetic of liquid/softgel L-T4 administered at breakfast** | Unclear | Undetected | Undetected | Not serious | Undetected | N/A | N/A | No | **Moderate** |
| **Compliance and quality of life of patients** | Unclear | Not serious | Undetected | Undetected | Undetected | N/A | N/A | No | **Moderate** |

N/A – Not applicable.

**Table 3S.** Characteristics of the included studies

| **Author et al. (Year)** | **Year** | **Country** | **Type of study** | **Participants' number** | **Participants' gender** | **Participants' mean age in years** | **Category of patients** | **L-T4 formulation** | **Comparison with tablet formulation?** | **Associated meal/beverage** | **Timing of L-T4 administration** | **Outcomes considered by the study** | **Main Findings** |
| --- | --- | --- | --- | --- | --- | --- | --- | --- | --- | --- | --- | --- | --- |
| Cappelli et al. (2014) | 2014 | Italy | Non-randomized trial | 54 | 89% F | Group A:  48.7 ± 11.1 Group B:  51.7 ± 12.6 | Hypothyroid patients without specified diagnosis | Liquid | No | Breakfast/Coffee | At breakfast | TSH, FT4 and FT3 serum levels | No differences observed in thyroid hormones concentration when L-T4 was taken at breakfast or 30 min before, for 3 and 6 months |
| Cappelli et al. (2016) | 2016 | Italy | Randomized crossover trial | 77 | 83% F | 45.4 ± 13.7 | Hypothyroid patients due to Hashimoto's thyroiditis or thyroidectomy for proven benign goiter | Liquid | No | Breakfast | At breakfast | TSH, FT4 and FT3 serum levels | No differences observed in TSH and thyroid hormones concentration when L-T4 was taken at breakfast or 30 min before. No effect of sequence of regimens, breakfast composition and/or concomitantly administered drugs |
| Cappelli et al. (2016) | 2016 | Italy | Non-randomized trial | 60 | 85% F | 47.7 ± 11.2 | Hypothyroid patients due to thyroidectomy for proven benign goiter | Liquid/Softgel | No | Breakfast | At breakfast | TSH, FT4 and FT3 serum levels | No differences in TSH levels, but FT3 and FT4 levels with the softgel capsule were significantly lower than those at with the liquid formulation |
| Cappelli et al. (2018) | 2018 | Italy | Cross-sectional | 320 | 85% F | 47.9 ± 15.6 | Hypothyroid patients without specified diagnosis | Liquid | Yes | Breakfast | At breakfast | MMAS-8 plus three further items to specifically evaluate medication preferences | Patients on L-T4 tablets forgot to take their medication more frequently and had difficulty in sticking to their treatment plan than those on liquid L-T4 treatment |
| Ducharme et al. (2022) | 2022 | Canada | Randomized crossover trial | 33 | 27% F | 38 ± 8 | Healthy volunteers | Liquid | No | High-fat, high-calorie meal | 15 minutes before a high-fat high-calorie meal | Serum T4 concentration, Cmax, tmax, AUC 0–48, AUC 0–72 | No significant difference in the pharmacokinetic properties of liquid L-T4 taken 15 and 30 minutes before a high-fat high-calorie meal |
| Giusti et al. (2014) | 2014 | Italy | Non-randomized trial | 59 | 85% F | 58 ± 13 | Hypothyroid patients due to total or partial thyroidectomy ± ablative radioiodine treatment | Liquid | Yes | Breakfast | 30 minutes, between 15 and 30 minutes, and <15 minutes before breakfast | TSH, FT4, FT3 serum levels, VAS | No change observed in TSH, thyroid hormones or thyroglobulin. A balanced breakfast containing less than 4 g of alimentary fibre did not interfere with L-T4 therapy |
| Guglielmi et al.  (2018) | 2018 | Italy | Non-randomized trial | 102 | 89% F | 49.1 ± 15.3 | Hypothyroid patients due to thyroidectomy or autoimmune thyroiditis | Liquid | Yes | Breakfast | At breakfast | ThyTSQ questionnaire, TSH, FT4 and FT3 serum levels | 66.6% of patients reported an increase in QoL after shifting from taking L-T4 30-60 minutes before breakfast to at breakfast. 10.7% of patients found the liquid formulation distasteful. No change in mean values of TSH, FT4, FT3, and of metabolic parameters |
| Marina et al. (2016) | 2016 | Italy | Ad-interim analysis of randomized trial | 21 | 67% F | Group S:  49.57 ± 9.36 Group L:  50.85 ± 8.93 Group LB: 53.42 ± 10.39 | Hypothiroid patients due to total thyroidectomy for thyroid cancer (before radio-iodine treatment) | Liquid | Yes | Breakfast | At breakfast; 30 minutes (or more) before breakfast | FT4 increase percentage | FT4 after 3 and 4 hours from the ingestion of 200 mcg liquid L-T4 is not influenced by meal and is comparable with that observed after tablet LT4 ingested while fasting |
| Morelli et al. (2015) | 2015 | Italy | Randomized crossover trial | 61 | 85% F | Group 1:  44.5 ± 9.5 Group 2:  48.7 ±10.0 | Hypothyroid patients due to Hashimoto’s thyroiditis,  total thyroidectomy or radioactive iodine treatment | Liquid | No | Breakfast | At breakfast; 10 minutes and 30 minutes before breakfast | TSH, FT4 and FT3 serum levels | Therapeutic equivalence between liquid L-T4 administration at breakfast or 10 min before breakfast |
| Pirola et al. (2014) | 2014 | Italy | Randomized trial | 20 | 10% F | Group T:  68 ± 5.8 Group L:  69.1 ± 5.1 | Hypothyroid patients due to total thyroidectomy | Liquid | Yes | Nutrison meal | With nutrison meal | TSH, FT4 and FT3 serum levels | Liquid L-T4 can be administered directly through feeding tube with no need for an empty stomach |
| Pirola et al. (2018) | 2018 | Italy | Non-randomized trial | 761 | 73% F | 46.9 ± 10.8 | Hypothiroid patients due to Hashimoto thyroiditis or total thyroidectomy | Liquid | No | Breakfast and drugs (excluding metformin) | At breakfast; 30 minutes (or more) before breakfast | TSH serum level | No differences in TSH observed whether L-T4 was ingested at breakfast or 30 min prior in a fasting state |
| Trimboli et al. (2020) | 2020 | Italy/Switzerland | Prospective | 121 | 83% F | median: 51 (IQR 42-62) | Hypothyroid patients due to Hashimoto or thyroidectomy for proven benign goiter | Liquid/Softgel | Yes | Breakfast | ≥30 minutes before breakfast; <30 minutes before breakfast | TSH serum level | Therapetic efficacy of liquid and softgel L-T4 is not influenced by correct or incorrect timing of administration in respect to breakfast |
| Vita et al. (2012) | 2012 | Italy | Non-randomized crossover trial | 8 | 88% F | 41-64 (range) | Hypothyroid patient due to Hashimoto’s thyroiditis and patients with benign nodules goiter or recurrence of nodules after thyroidectomy | Softgel | Yes | Coffee | 60 minutes or ≤5 minutes before coffee | TSH serum levels | Coffee influenced L-T4 pharmacokinetics minimally |

TSH – thyroid stimulating hormone ; FT4 - thyroxine; FT3 - triiodothyronine; L-T4 - levothyroxine; MMAS-8 - Eight-item Morisky Medication Adherence Scale; Cmax - maximum observed concentration; tmax - time of observed maximum concentration; AUC 0-48 - area under the concentration–time curve from time 0 to 48 hours; AUC 0-72 - area under the concentration–time curve from time 0 to 72 hours; VAS – visual analogue scale; QoL – quality of life
